# A strategy to what end? “The strategy of model building in population biology” in its programmatic context

**DOI:** 10.1007/s40656-024-00646-2

**Published:** 2024-11-18

**Authors:** Zvi Hasnes-Beninson

**Affiliations:** Inter-University PhD Program in the History and Philosophy of the Life Sciences, Cohn Institute for the History and Philosophy of Science and Ideas, Tel-Aviv University, Tel Aviv-Yafo, Israel

**Keywords:** Modeling complex systems, Population biology, Richard Levins, Fundamental and applied science, Environmental granularity, Natural resource management, Heterogenous environment, Fitness, Model intelligibility

## Abstract

“The Strategy of Model Building in Population Biology” published by Richard Levins in 1966 has been cited over 2500 times. For a paper concerned with modeling approaches in population biology a surprisingly large part of the attention. The Strategy received comes from history and philosophy of biology, and specifically from accounts on model and model formulation. The Strategy is an unusual paper; it presents neither new data nor a new formal model; at times it reads like a manifesto for some modeling approach, without specifying which broader program that approach intends to support. When these peculiarities of The Strategy are even mentioned, the philosophical literature tends to explain them away by invoking Levins’ Marxist commitments. In contrast, I argue that those peculiarities can be explained by examining the programmatic purpose of the paper; starting from his doctoral work, Levins was trying to establish a research program meant to account for the relations between fitness and environment in different terms than the prevalent lock-and-key view. My paper brings that program back to the discussion, explains its relation to competing approaches and examines Levins’ approach to modeling in light of that context

## Introduction

According to Google Scholar, as of October 2024, the paper “The Strategy of Model Building in Population Biology” (Levins, 1966), written by the ecologist and population geneticist Richard Levins (henceforth: The Strategy), has been cited over 2800 times. For a technical paper concerned with modeling approaches in population biology, a surprisingly large part of the attention The Strategy received came from philosophers of biology. This became prevalent during the late 1980s, with the lion’s share of attention coming from philosophical accounts of models and model-formulation.

The Strategy is an unusual paper; it presents neither new data nor a new formal model; its use of terminology is uncommon; and at times it reads like a manifesto for some modeling approach, without specifying which broader program that approach intends to support. Given the scarcity of citations in the five years that followed the publication of The Strategy, the overall impression is that Levins did not write on behalf of some broad movement in ecology, and it is unclear who were Levins’ intended addressees and who (if any) the other participants in that program were.

When these peculiarities of The Strategy are mentioned at all, the philosophical literature tends to explain them away by invoking Levins’ Marxist commitments. For example, in an otherwise enlightening paper on The Strategy, Jay Odenbaugh was concerned with Levins’ treatment of modeling desiderata, specifically with the term “contradictory desiderata”. When addressing that point, Odenbaugh writes as follows (Odenbaugh, 2006, p. 618):

It is true that Levins uses the phrase “contradictory desiderata” which suggests there might be a tension between model properties alone. However, first it is important to note that in the very same paragraph Levins mentions the “contradictory demands” between a complex nature and human minds. Second, Levins is a Marxist and Marxists use the term ‘contradiction’ in a different sense than simply a proposition that is logically inconsistent. [Q1]^1^

Odenbaugh is correct on both accounts. First, it is easy to establish that Levins was a committed Marxist. Levins was born in 1930 in Brooklyn, studied mathematics and agriculture for his undergraduate diploma (graduated at Cornell University). Levins was so outspoken about his Marxist commitments during his time at Cornell that he was subjected to an FBI investigation,^2^ and left the United States for over five years to live in Puerto Rico before returning to the US for his doctoral work. Second, the Marxist use of the term ‘contradictory’ is indeed different from the standard use. Marxists tend to use ‘contradiction’ in relation to a process (as opposed to a proposition) in which the outcome undermines the conditions of possibility. From the final chapter of *The Dialectical Biologist*, it seems that Levins held a notion of ‘contradiction’ along similar lines (Levins & Lewontin, 1985, pp. 273-275).^3^

However, I find three shortcomings with explanations of the peculiarities of The Strategy that evoke Levins’ Marxism: first, precisely because these commitments are so easy to establish, everything in Levins’ intellectual legacy could (in principle) be attributed to them, and they do not reveal anything specifically related to The Strategy. Second, assuming that even the most devoted Marxists are not all painted in the same shade of red, merely referring to Levins’ Marxism does not explain much without clarifying the version of Marxism that he adhered to. By the time of writing of the present paper, such work has not been done. Finally, I am not certain that evoking his Marxist commitments enlightens us about Levins’ scientific work. As [Q1] demonstrates, Odenbaugh offers two ways to treat Levins’ use of the term “contradictory desiderata”, but as will be discussed later in the paper, the first way, namely, the *contradictory demands between a complex nature and human minds* allows a coherent reading of Levins. The second treatment ascribes to Levins an overly convoluted approach to model formulation, one that seems implausible to begin with, and the historical record does not support Levins’ adherence to such an approach.

Thus, the current paper refrains (as much as possible) from invoking Levins’ Marxism when addressing the peculiarities of The Strategy. The purpose of the present paper is to understand Levins’ general approach to modeling by contextualizing The Strategy in the research program it meant to serve, namely, population biology. Put bluntly, I argue that The Strategy was meant to formulate the modeling approach to be used in population biology, and accordingly, its peculiarities can be explained by examining that context. For that purpose, it should be noted that the current paper will not treat The Strategy as a stand-alone work; instead, The Strategy will be treated as an outline of an unfolding program. While some of the works in that program predate The Strategy−including Levins’ own doctoral work, which was published as a series of paper starting with (Levins, 1962)−much of the program was developed after The Strategy. Put differently, contextualizing The Strategy in population biology requires addressing works from that program that were published after 1966.

The paper will proceed as follows: the second section will describe The Strategy, elaborate on some of the peculiarities of that paper, and explain why reading that paper alone does not solve these peculiarities. To be clear, modeling is indeed a central theme in The Strategy, but it is not the only one. Moreover, since the different themes in The Strategy are interrelated, untangling the peculiarities of The Strategy involves highlighting aspects of that paper that did not receive the same level of attention as model-formulation. The third section will focus on the relations between fundamental and applied science; Levins’ construal of these relations will accompany all the sections that follow. The fourth section is where the contextualization takes place; the starting point for the discussion will be the ecological and environmental context of The Strategy. The paper argues that The Strategy was informed by an ongoing effort (both theoretical and practical) to confront the problems associated with environmental degradation. Finally, the fifth section returns to The Strategy and elaborates on the modeling approach in that paper based on the contextualization.

## The strategy of model building in population biology: an overview

The Strategy begins by describing the emergence of population biology as the coming together of population genetics, population ecology, and mathematical biogeography. Prior to that, these fields had developed with *different assumptions and techniques* (Levins, 1966, p. 421). The assumptions could be complementary such that what is held constant in one field (e.g., the environment in population genetics) is allowed to vary in another (population ecology) and vice-versa. The new unified field of population biology had to deal simultaneously with *genetic*,* physiological*,* and age heterogeneity within species of multispecies systems changing demographically and evolving under the fluctuating influences of other species in a heterogenous environment* [Q2]. The immediate difficulty that The Strategy was meant to address was how do deal with such a complex system.

Levins began by rejecting a one-to-one modeling approach to such a system, rendering it as a *naïve*,* brute force approach*. Such an approach would have entailed the formalization of a very large set of partial differential equations with time lags that had to be solved simultaneously; measuring an even larger number of parameters, solving the equations to get numerical predictions, and comparing these predictions to observations. Levins noted that this approach encounters some difficulties: first, there are too many parameters to measure, not all of them sufficiently defined, and even those that are well-defined may require a long time to measure. Second, the equations are not analytically soluble; finally, even if the equations are soluble, the solution may be meaningless.

Thus, Levins required that the models be simplified while preserving the essential features of the problem. The legitimacy of such simplification depended *not only on the reality to be described but also on the state of the science* (Levins, 1966, p. 422). Levins then stated three desiderata to be maximized (generality, realism, and precision) for achieving the overlapping but not identical modeling goals of understanding, predicting, and modifying nature. However, since maximizing all three desiderata would result in unmanageable models, and since unmanageable models cannot achieve the stated modeling goals, one desideratum has to be sacrificed in order to maximize the other two. Thus, we get three modeling strategies:

1. Sacrifice generality to maximize realism and precision. This strategy reduces the number of parameters, measures the parameters of interest accurately, solves the equations numerically, and generates precise and testable predictions. However, it is only applicable to particular situations.
2. Sacrifice realism to maximize generality and precision. The equations used in this strategy are general and precise results could be obtained, but the modeling assumptions are not realistic. There are expectations from the assumptions within this strategy, most notably, that the unrealistic assumptions will cancel each other out. Thus, the strategy is hoped to increase overall realism.
3. Sacrifice precision to maximize realism and generality. This is the approach advocated in The Strategy (Levins, 1966, p. 422): *since we are really concerned in the long run with qualitative rather than quantitative results (which are only important in testing hypotheses) we can resort to very flexible models*,* often graphical*,* which generally assume that functions are increasing or decreasing*,* convex or concave*,* greater or less than some value instead of specifying the mathematical form of an equation*. [Q3]

However, this modeling strategy encounters the following problem: while type-3 models maximize realism, they also have artificial assumptions, and it is thus doubtful whether a result of such model derives from maximizing realism or from the details of the simplifying assumptions made. This is the problem that closes the first part of The Strategy, and to cope with it Levins offered the following solution (Levins, 1966, p. 423):

Therefore, we attempt to treat the same problem with several alternative models each with different simplifications but with a common biological assumption. Then, if these models, despite their different assumptions, lead to similar results we have what we can call a robust theorem which is relatively free of the details of the model. Hence our truth is the intersection of independent lies. [Q4]

The second part of The Strategy elaborates on the issue of theorem robustness and begins with an example of a robust theorem, namely, that *in an uncertain environment*,* species will evolve broad niches and tend toward polymorphism*. To substantiate this theorem, Levins deployed three models that he had used in publications prior to The Strategy. The technical details of these models are not important for present purposes. The more relevant issue is the general point that The Strategy makes in the second part, namely, that the models were meant to address different aspects of the theorem, and to do so made different assumptions about their target system; yet despite these differences, the models produced similar results about the qualitative trends of the system. The similarity in results despite differences in assumptions meant that together, the models provide a higher level of confidence in those results than any single model could have achieved individually (Levins, 1966, p. 425).

While the second part of The Strategy offers robustness as a mechanism to dealing with unrealistic assumptions (prevalent even when maximizing realism), the third part is concerned with a feature of general models, namely, that the variables in their target systems could be numbered in thousand or more. This amount should be reduced so that the models will be manageable, and this reduction is done by a process of abstraction whereby many terms enter into consideration as higher-level entities; for example, *all the physiological interactions of genes in a genotype enter the models of population genetics only as part of “fitness”* (Levins, 1966, p. 428). Levins dubbed these higher-level entities as “sufficient parameters” and their number is greatly reduced compared with the target system’s characteristics. Sufficient parameters must be suitable for a given level of analysis, with “suitability” being a function of the ability of the sufficient parameters to contain the important information about events on that level. Sufficient parameters are the result of many (lower-levels variables) to-one (higher-level entity) transformation. This is the source of their utility, but it is also a source of information loss; first, the many-to-one transformation is a source of imprecision. Second, once the transformation is made, it is not possible to locate the low-level parameter which is the source of the higher-level imprecision. In order for a general representation of nature to be robust, a relation between the lower-level and higher-level entities must be established (Levins, 1966, p. 429):

It becomes necessary to supplement our theorem with some subordinate models which explain how to go from “uncertainty” to the components of the environment and biology of the species in question. Thus general models have three kinds of imprecision: […] Hence, the general models are necessary but not sufficient for understanding nature. For understanding is not achieved by generality alone, but by a relation between the general and the particular. [Q5]

In its final part, The Strategy returns to generalizing. It stresses that models leave out most elements of their target systems and are thus not verifiable directly by experiment. Instead, the validation of a model depends on its ability to *generate good testable hypotheses relevant to important problems* (Levins, 1966, p. 430). Moreover, since models are technically restricted to considering a small number of elements at a time, a theory is conceived as a cluster of models that relate to each other in some ways. The Strategy ends by stressing that the necessary use of cluster of models derives from the need to cope with contradictory requirements from models, namely, the complexity of the target system on the one hand, and our cognitive limitations on the other.

### Why is the strategy unusual?

As seen above, The Strategy seems to have two objectives. The first goal is to generally discuss modeling strategies. Within that general objective Levins enumerated three modeling goals (understanding, predicting, and modifying nature) that overlap with the modeling desiderata (generality, realism, and precision). Having recognized the futility of unmanageable models on one hand, and the inability to produce a manageable model while maximizing all the desiderata on the other, Levins concluded that one desideratum must be sacrificed in order to produce a manageable model that maximizes the other two. Hence, we get different modeling strategies. That general objective has received most of the philosophical attention; notable works in that vein include, but are not limited to, (Gelfert, 2011; Godfrey-Smith, 2006; Isaac, 2013; Justus, 2006; Matthewson, 2011; Odenbaugh, 2003; Orzack, 2005; Plutynski, 2006; Weisberg, 2006a, b; Wimsatt, 2006, 2007).

While modeling is indeed a central aspect of The Strategy, it is not the only one. By his own admission, Levins preferred a modeling strategy that sacrifices precision in order to maximize generality and realism. The second objective of The Strategy is to elaborate on this modeling strategy. As discussed above, the second and third parts of The Strategy are a detailed and technical discussion of robustness, as well as on the use of sufficient parameters. While the philosophical literature tends to assume (at least implicitly) that The Strategy is about modeling in the abstract, the second objective poses some difficulties to that assumption. For example, if Levins intended to discuss modeling in the abstract, why did he not suggest an exhaustive and extensive list of desiderata? And why did he not define the desiderata that he did enumerate?

However, the difficulty that seems most pressing to interpreting The Strategy as an abstract discussion of modeling is the following: given Levins’ admission that models are partial representations based on artificial assumptions and are thus literally false, why did he favor type-3 models? Although favoring one type of modeling approach is not inconsistent with theoretical writing about model-formulation,^4^ when such predilection appears in a theoretical work, it usually highlights the epistemic or methodological superiority of the favored approach compared with the alternatives.

Levins explained his preference by alluding to the flexibility of qualitative models, stating that quantitative results *are only important in testing hypotheses* (Levins, 1966, p. 422), but then acknowledged that type-3 models do not solve in-principle issues such as artificial assumptions (Levins, 1966, p. 423). Type-3 strategy suited Levins’ modeling purposes, but he did not set out to substantiate that modeling approach as superior to the alternatives; instead, he dedicated a lengthy discussion on how this approach could be deployed technically. Moreover, Levins’ solution for conceptual difficulties associated with modeling (such as artificial assumptions) involved the use of cluster of models, not any type of modeling strategy. Put differently, when The Strategy engages with conceptual issues, it does not show any reason to favor one modeling strategy over another.

Before continuing, I would like to clarify that it is not my intention to imply that the literature concerning The Strategy is based on a misreading, and it is certainly not my claim that attempts to draw general conclusions about modeling from that paper are inappropriate. Rather, I claim that The Strategy only seems unusual when the paper is conceived as an abstract discussion on modeling, and that some of the difficulties are resolved when its programmatic context is considered. Hence, the current paper assumes that The Strategy was *not* meant to be a theoretical discussion on modeling in the abstract. Instead, I argue that a central goal of The Strategy was to outline a methodology for an emerging research program; that that program tried to address concrete questions; and that we can gain better understanding of The Strategy by unravelling those concrete questions. I will return to the modeling-related aspects of The Strategy after contextualizing that paper.

### Generality, realism. Specificity?

The claim that The Strategy was not meant to be a work about modeling in the abstract does not entail that The Strategy cannot be used in abstract discussions on modeling. Quite the contrary, the relevance of the 1966 paper for general discussions on modeling has been amply demonstrated. Rather, the argument here is that focusing exclusively on the lessons that could be learned from The Strategy in such discussions runs the risk of ignoring other lessons that The Strategy alludes to. In the current section I focus on the relations between the desiderata that constitute Levins’ favorite modeling strategy, i.e., realism and generality.

In an assessment paper from 1993, Steven Orzack and Elliott Sober point out that Levins did not provide a definition to any of the desiderata enumerated in The Strategy, *nor does he provide an argument for why they are mutually antagonistic* (Orzack & Sober, 1993, pp. 533-534). To be precise, Levins did not argue that the desiderata are antagonistic; in fact, he did not even argue that the three desiderata cannot be maximized at the same time. Levins’ argument was that a model resulting from such maximization will be unmanageable. While Orzack and Sober are correct that Levins did not provide a definition to his desiderata, in his response to their assessment Levins explains that choice (Levins, 1993, pp. 547-548):

The formal mode of thought prefers sharply separated disjunct categories and has indeed encouraged the long list of false dichotomies that have plagued our biology: heredity versus environment, physical versus biotic control of population abundance, internal versus external determination, random versus deterministic processes, and equilibrium versus non-equilibrium systems. [Q6]

The value of The Strategy does not depend on accurate definitions of the concepts it uses; to the extent that *the formal mode of thought* demands precise definition of general concepts such as “generality” or “realism”, this mode of thought is (at the very least) inconsistent with the practice of various scientific disciplines. However, although precise or exhaustive definitions should not be seen as a requirement, when communicating abstract ideas such as modeling desiderata, a working definition of the terminology could be beneficial. Levins’ insistence not to define his terminology even in his reply to Orzack and Sober should be noted.

The exchange between Orzack and Sober and Levins highlights a specific issue concerning the relations between “generality” and “realism”. In Orzack and Sober’s view, the relations between generality and realism are not fixed, but these desiderata stand in a continuum; thus, for example, the difference between a realistic and a general model could be that the realistic model is instantiated,^5^ whereas the general model is uninstantiated (Orzack & Sober, 1993, p. 536). For Levins, on the other hand, it is not realism that stands in continuum with generality, but specificity (Levins, 1993, p. 552):

As we make the model more specific we are losing generality in two ways: each step that adds new variables or new relations among them excludes those cases in which these particular variables or relations are not operative. And once we have introduced new variables we may have to say something about the mathematical forms of their interactions, the exact equations, or the magnitudes of their parameters. The last stage in specification is instantiation. [Q7]

Relying on later works in an attempt to interpret earlier works runs the risk of reading views into the earlier work that were not on the author’s mind when publishing it. To be explicit, it seems plausible that in 1966 Levins had not thought in terms of “specificity” and was only pushed to considering the issue by Orzack and Sober’s 1993 paper. However, the circumstances of the 1993 argument might shed light on the 1966 paper. In 1993 Orzack and Sober suggested that realism and generality are two ends of the same continuum; this view seems to entail that maximizing both realism and generality is contradictory (in the regular use of the word). Realizing that his favorite approach to modeling could be seen as contradictory, Levins evoked in his response specificity as the polar opposite to generality, while conceptualizing realism as an entirely separated category from generality. The important point was the rejection of the notion that generality and realism stand in a continuum; the term “specificity” was used for that purpose and was not important on its own. From the text of The Strategy, it seems quite plausible that already in 1966 Levins had conceptualized generality and realism as independent vectors that could in principle be maximized together.

## Levins’ views on the relations between theoretical and applied science

Nicholas Wade was the editor of the Journal *Science* between 1972 and 1982; in the 180th volume of the journal, from May 1973, Wade published an article in which he pointed out some social implications of the industrialization of American agriculture, a process that began in the early 1940s and (according to Wade) was encouraged by the USDA (Wade, 1973). In the article, Wade suggested that these social implications should be studied by professional social scientists and called for public funding of that research. In the 181st volume of *Science* from August 1973, Levins published a response (Levins, 1973a). In his response, Levins argued that the problems in agricultural research that Wade had highlighted were a manifestation of a broader (and every bit as problematic) phenomenon of separation between fundamental and applied research.

Although The Strategy predates Levins’ response to Wade by several years, discussing Levins’ views on the relations between fundamental and applied research early in the current work is important for two reasons: first, the programmatic context of The Strategy was an ongoing project before and after its publication in 1966. That context highlights that Levins adhered to a view that fundamental and applied research inform each other in important ways. The current section will show that this view is implicit in some of Levins’ earlier works, such that it is temporally closer to The Strategy than 1973. Second, the view about the relations between fundamental and applied research plays an explanatory role in later sections, so it is best to clarify the issue in advance.

On the applied side of things, Levins recognized an anti-theoretical bent in agriculture that took for granted the existing structure of related industries and that was reinforced by profit-oriented priorities. On the theoretical side, Levins lamented the bias of research institutions against applied work, agriculture included. Levins suggested several ways to bridge the gap between fundamental (ecology) and applied (agriculture) research: as for the interest of agriculture in fundamental research, Levins stressed that in the absence of profound understanding of the systems involved, the long-term results of that practice were likely to be counter-productive to its own goals. On the theoretical side of things, Levins highlighted that agriculture as a discipline was mostly neglected by theoreticians, who left the field to be dominated by practical concerns that do not necessarily relate to providing enough nutrition to the population. Levins was concerned with this neglect since the ecological communities associated with cultivated fields are historically new and as such *these systems are ideal for the study of dynamic aspects of evolutionary ecology*. (Levins, 1973a, p. 523). On top of that concern, Levins thought that insight from fundamental systems (tropical rain forests) are applicable to applied systems (cultivated fields), and that the latter could be studied using the same methodological approach as the former such that applied research could support long-term programmatic objectives of theoreticians (Levins, 1973b, p. 523):

The unifying principle of a management goal tends to break down disciplinary boundaries and should create an integrated population biology in which community structure, physiological responses, migration-extinction patterns, bioclimatology, and natural selection can be studied together. The inner logic of population biology requires increasingly that we study complex systems as wholes. A practical long-range goal may get us to do so. [Q8]

This quote highlights a way in which applied research could lead to more fundamental research, namely, that research done for pragmatic reasons leads to certain theoretical questions. Thus, the applied community is seen as a particular, more approachable, case that serves as a gateway to studying the fundamental, more complex, community. Moreover, this relation works both ways; the fundamental research aims to achieve practical goals, most notably, the management of nature. The issue of a management goal is relevant to later parts of the current paper.

To be sure, the paper from 1973 is the earliest work I found where Levins fully articulated his position regarding the appropriate way to conceive the relations between fundamental and applied research in ecology; he would repeat that view throughout his career.^6^ However, in the 1973 paper Levins enumerated ten areas of study that he considered to be both agriculturally relevant and fundamental, and believed that both sides (applied and fundamental) could benefit from further research. The important point for the present discussion is that Levins had worked on the theoretical aspects of at least six of the ten areas prior to 1973. Regarding some of those areas, Levins had identified their relations to either agriculture or to other applied issues already in the original publication. For example, concerning integrated population biology, Levins co-authored the following in 1970 (Sammeta & Levins, 1970, p. 470):

Genetics and ecology come together in another way in the increasingly important applied areas of resource management, integrated pest control, and plant and animal breeding. Organisms of economic importance are under long term, wide ranging surveillance uninfluenced by changes of personnel. Therefore they are a rich source of information on seasonal and irregular fluctuations, sensitivity to environment, geographic variation, and rapid changes under new agronomic conditions. Whole categories of genes are identified in these groups only by interactions with host genotypes. [Q9]

Another example relates to feedback dynamics within a community that results in a species selecting itself into a local extinction (first item on the list in the 1973 paper), for example by modifying the environment to the point where it cannot support the local population. Such a dynamic could be deployed as a method of pest control, and in a study from 1970 that analyzed different ways for such a dynamic to emerge, as well as its possible effects on different levels of the community, Levins wrote the following (Levins, 1970a, pp. 78 − 79):

Extinction is important in two other connections. It is fundamental to any theory of pest control, and it is in the field of economic entomology that it is possible to study real population over long periods of time and wide areas. Therefore a satisfactory theory of extinction will make it possible to incorporate a number of applied subjects into general ecology. [Q10]

Finally, regarding the distribution of pests and predators over a region within the framework of extinction-migration theory of quantitative biogeography (third item on the list in the 1973 paper), Levins had engaged with the applied aspects of the extinction-migration theory in a paper from 1969, as part of an attempt to establish a method of pest control that does not rely of pesticide (Levins, 1969). Put differently, the very motivation behind that paper was Levins’ concern with agricultural considerations.

While it is possible to show that Levins harbored similar concerns regarding other items of the 1973 paper’s list, the general point is clear: from a relatively early stage, Levins held the view that fundamental and applied research should not be seen as dichotomous but as being in a dialectical relation where each side informs the other. This relation relates to a modeling goal enumerated in The Strategy, namely, modifying nature. I will elaborate on that goal later in the paper; for now, it is important to stress that the modeling goal of modifying nature seems to indicate that Levins considered modeling as a practice that, at least potentially, enables an intervention upon nature.

## The programmatic context of the strategy

### Background

In the wake of military experiments in nuclear weapons during the 1950s, ecologists became increasingly concerned with the fact that humans, and especially industrialized societies, affect their natural surroundings. Similarly concerning was Rachel Carson’s *Silent Spring*, published in 1962; while the effects (environmental or otherwise) of nuclear weapons are conspicuous, *Silent Spring* demonstrated that a far more mundane practice, namely, the widespread use of pesticides for agricultural purposes, could also entail dire long-term environmental effects (though not as immediately striking). Ecologists have never been oblivious to the dangers of environmental degradation, even before the aforementioned nuclear experiments. An overview of the history of ecology and its relations to environmentalism prior to the 1960s exceeds the scope of the current paper; however, (Kingsland, 2005; Mitman, 1992) offer an introduction to these issues.

While awareness of environmental degradation and the concern that humans themselves will ultimately be the victims of that degradation were not new in the 1960s, some related issues became increasingly pressing as a result of the nuclear experiments of the 1950s and Carson’s *Silent Spring*. Issue 14(7) of the journal *BioScience* was dedicated to some of the ecological challenges of the mid-1960s, and the future of ecology as a discipline in light of these challenges. Among the papers included in *BioScience* 14(7), three recurring concerns are relevant for present purposes: first, that increased population means that human societies affect the environment to a greater degree (Blair, 1964; Cole, 1964; Platt et al., 1964). Second, that improved technology means not simply influence but control (Blair, 1964; Odum, 1964). Finally, that increased control means that policy for management of natural resources is not only possible but required, and that ecology as a discipline can help formulate informed policies for such purposes (Blair, 1964; Cole, 1964; Kuchler, 1964). Given the greater level of influence of industrialized societies upon the environment, and the risk of degradation, ecologists were under an imperative to assume a more active role in the public aspects associated with natural resources management.

### The rejected program

The approach that emerged as the dominant research program to coping with the theoretical and practical challenges facing ecology during the late 1950s and early 1960s was ecosystem ecology (Palladino, 1991, p. 224). Ecosystem ecology applied principles of systems analysis to study population-level phenomena. Within that program, ecological research consisted of four consecutive steps (Palladino, 1991, p. 228):

1. Catalogue all the factors implicated in the determination of a given ecological phenomenon, regardless of their biological or physical nature; put differently, those factors are to be treated as black boxes.
2. Measure the effect of each black box on population density over a given period and analyze the various measurements statistically. This analysis would allow both the identification of the components most responsible for the observed outcome, and the simplification of the initially complex model.
3. Formulate mathematical rules that would allow calculation of the numerical relations between the remaining black boxes.
4. Analyze the resulting mathematical formulae in an attempt to define their meaning in terms of biological phenomena and then break them down into a new subset of black boxes.

Proponents of ecosystem ecology believed that ecology must develop a unified theory of the ecosystem, described in precise mathematical and statistical terms, if the field was to be of any practical use. To that end members of the program implemented methods of general system theory and especially thermodynamics to ecology. In addition, these advocates held that such a theory must be holistic, not reductive (Odum, 1964, p. 14). Levins, a long-term adversary of reductionism, accepted the holistic approach, and agreed that population is the appropriate level of analysis of ecological systems. However, Levins rejected the managerial ethos implied in ecosystem ecology’s version of holism. Levins was skeptical of this ethos in part due to his own conception of holistic view: the interconnectivity of different parts of ecological communities makes them highly complex. Complexity, as a feature of a system, severely restricts our ability to predict the long-term behavior of the system. As seen in [Q3], Levins’ preferred approach to coping with this limitation is to use a qualitative model that only predicts a general trend of a system (Levins, 1966, p. 422).^7^

Ecosystem ecologists advocated informed ecological interventions based on accumulated knowledge. The problem was that the relevant knowledge at the time had been accumulated only for a few decades. Levins was skeptical that managerial decisions based on knowledge accumulated in that time span could lead to better results for the communities to-be-managed than the evolutionary processes that shaped them in the first place. Put differently, Levins’ notion of holism held a different standard concerning the meaning of “informed”. However, when facing environmental degradation and the associated risks, a devoted environmentalist such as Levins could not have refrained from action by adhering to standards of verification that render any intervention “uninformed”.

In later years, Levins claimed that for him the mutual influence of scientific and political activity had made more sense than the separation between the two (Levins, 2007). Levins’ personal archive seems to support that claim, at least regarding environmentalism. Early indications of Levins’ commitment to environmentalism were manifested both in his political activity (Levins & Lewontin, 1971), and in his previous scientific work (Heatwole et al., 1963; Levins & Heatwole, 1963; Rolle et al., 1964). Being committed to environmentalism, while rejecting the type of managerial ethos he ascribed to ecosystem ecology, Levins participated in a research program meant to re-examine fundamental concepts in ecology and evolution. While ecosystem ecologists attempted to formulate a grand synthesis of ecology (Odum, 1964, pp. 14–15), the program that Levins adhered to considered the ability to generate testable predictions as the most important goal, so that the models used for informed decision-making could always be compared with the reality outside the model. That program sought to generate such predictions through meticulous observations in small parts of the system under analysis. Kingsland elaborates on the types of problems addressed by that program (Kingsland, 1995, pp. 187-188):

The analysis which followed looked at two kinds of problems. First, given that niches were fixed, under what conditions could species coexist? Second, how would natural selection affect the position and breadth of the niche itself? From these questions they [Levins and MacArthur] tried to predict the situations under which character divergence or character convergence would occur. [Q11]

These were the problems that population biology set out to resolve, and The Strategy was meant to be a methodological outline for that research program. In this sense, The Strategy was indeed a paper concerning modeling, but not in the abstract decontextualized sense; rather, it was concerned with the kind of modeling required for the specific purposes of population biology. That program, as envisioned by MacArthur and Levins was predicated to a large extent on their novel perception of niche and fitness, and specifically on the relations between the term “fitness” and features of the environment.^8^

In 1967 Syracuse University hosted an international symposium on population biology. The proceedings of that symposium were published as (Lewontin, 1968), and the impression that they give is that a central motivation for that program was a general dissatisfaction among the participants with the Central Dogma of molecular biology which postulates that *the transfer of information from nucleic acid to nucleic acid*,* or from nucleic acid to protein may be possible*,* but transfer from protein to protein*,* or from protein to nucleic acid is impossible* (Crick, 1958, p. 153). Specifically, the dissatisfaction was with the implication of the Central Dogma on the Modern Synthesis, namely, that it allows evolutionary biologists to treat the unit of selection and the unit of inheritance as if they were one and the same. This treatment raises a multitude of conceptual and empirical difficulties. For example, in the same volume George L. Stebbins writes (Stebbins, 1968, p. 18):

Failure to appreciate the complexity of gene action in development has been responsible for much confused thinking about the action of natural selection. The problem lies in the fact that the selective action of the external environment, by its very nature, can operate only upon individual phenotypes, through their survival, death, or differential rate of reproduction. Evolution, however, must be measured not in terms of altered phenotypes, but as a function of changes in the composition of the gene pool. Between the act of selection and the response of the population lie all the complexities of gene action in development. [Q12]

While Stebbins addressed the issue of separating between the unit of selection and the unit of inheritance from a developmental point of view, other participants approached the same issue differently. However, from the perspective of evolutionary theory, the separation between the units of selection and of inheritance also raises a conceptual difficulty, namely, if the units are not one and the same, it is not clear whether predicting selective advantage is even possible. This has critical implications on the term “fitness” and its role in evolutionary theory. The closing paper in the same volume (Slobodkin, 1968) attempts to articulate a predictive theory that does not rest upon the unification of the unit of selection with the unit of inheritance.

### The ecological context

The full title of The Strategy explicitly refers to the domain in which the various modeling strategies discussed in the paper apply, namely, “in population biology”. However, by 1966 at the time The Strategy was written, “population biology” was not yet fully formed. To be sure, the fields that were supposed to integrate into population biology had existed before The Strategy and Levins refers to various works in those separate fields; however, population biology did not yet exist as an integrated synthesis. Levins admitted as much in The Strategy, where population biology was regarded as a field that still needed to be established so that the causal factors ignored by its individual fields (population genetics, population ecology, and mathematical biogeography) could be properly addressed (Levins, 1966, p. 421).

Levins did not write his comments on population biology as a neutral observer anticipating the emergence of a new research program, but as an active participant in the efforts to integrate the separate fields comprising population biology into a unified synthesis. While some aspects of population biology had been addressed in Levins’ doctoral work, published as (Levins, 1968b)^9^ and others were addressed at roughly the same years as The Strategy (Levins, 1968c), certain questions do not seem to have occurred to Levins until the early 1970 (Levins, 1974).

From an ecological point of view, the motivation for population biology was to explain the interrelations between organisms and their environment in evolutionary terms (MacArthur, 1968). Accordingly, the treatment of ecological niche was central for population biology, and two points needed to be addressed, namely, the competitive exclusion principle [CEP] and Hutchinson’s definition of niche. CEP means that co-existing species cannot occupy the same niche (Justus, 2021, p. 18). For that principle to have any meaning, we must have some definition of niche; Hutchinson conceptualized the environmental factors (biological and non-biological) that affect a species as axes that delineate an abstract *n*-dimensional space. Within that space, niche is defined as the area where the species is able to persist (in the absence of competition). The subset of this space where the species has positive fitness is the *fundamental niche* of that species (Justus, 2021, p. 30). When competition is present, the species occupies a smaller portion of the fundamental niche, which is called the *realized niche* (Kingsland, 1995, p. 182).

The conceptual difficulties of these definitions are anything but trivial; for example, if the concept of realized niche depends on the presence of a species occupying it, then the meaning of the term “niche” seems to reduce to “part of the environment occupied by species” (Justus, 2021, pp. 32 − 33). This amounts to a circular definition whereby the fitness of a species depends on it occupying its fundamental niche, while the niche depends on a species occupying it. Another difficulty derives from discrepancies between the predictions that stem from the definitions above and the empirical findings. For example, Hutchinson’s definition predicts that realized niches will be discrete, but MacArthur’s data, gathered from his observations on warblers, suggested that niches were continuous (Kingsland, 1995, p. 186).

Finally, the term niche, as developed by Hutchinson (and earlier researchers) assumed that the environment remains unchanged and that the causal relations between the environment and the organism are unidirectional from the former to the latter (Justus, 2021, p. 52). In order to formulate an evolutionary ecology, those assumptions had to be replaced. A crucial term in the framework that Levins and MacArthur suggested is “grain”; Kingsland describes the concept of grain and the way it affects fitnesses (Kingsland, 1995, p. 195):

Originally developed by MacArthur and Levins, the concept of grain was a way of relating resources to the size and the search strategy of the predator. If resources were mixed in such a way that the predator found them in the proportion in which they occurred in the environment, then they were said to be “fine-grained”. If, on the other hand, the predator could find one resource in more than its natural proportion, the resource was “coarse-grained”. The purpose of their analysis was to show that diversity depended not only on the number of resources and predators, but also on the “graininess” (relative size and distribution) of resources. [Q13]

The category of environmental granularity was introduced to replace the stability assumption made by the term “niche”. While niche takes the environment as constant, the very definition of granularity is relational such that graininess measures how rapidly the environment changes within the lifetime of an individual. Two points should be clarified in this regard: first, that the rapidity of change is not strictly defined in either spatial or temporal terms; the succession of environments can take place either in time or in space. Second, there is no universal measure of graininess; the same environment could be fine-grained for one species and coarse-grained for another (MacArthur, 1968, p. 161). Taken together, the level of environmental granularity is a parameter that affects the genetic variation in a population, such that the more fine-grained the environment, the less likely it becomes that genetic variation will occur.

Within Levins’ attempt to outline what kinds of changes in the environment may lead to what kinds of plasticity,^10^ “grain” became central for accounts if environmental heterogeneity, and Levins’ 1968 book, *Evolution in Changing Environments* can easily be described as an exploration of the evolution of responses to changing conditions. Levins recognized that, *ceteris paribus*, a heterogenous environment poses challenges that a stable environment does not, simply by virtue of being heterogenous. In addition, Levins recognized that in order to sustain life, environmental fluctuations must be within certain limits (Levins, 1968b, p. 35).

Under conditions of environmental stability, fitness is understood as a process whereby the organism is required to optimize its morphology to challenges and restrictions that the environment poses. However, a changing environment means that there is no single point to optimize to; rather, there are different environmental conditions, each posing its own minimal requirements for survival. Moreover, the causal relations between organism and environment are not unidirectional if the organism’s behavior affects its environment. This does not mean that Levins adhered to niche construction during the 1960s; in a paper published after his dissertation, Levins claimed that ecology as a discipline lacked the appropriate mathematical language for a theory of niche construction (Levins, 1968c). Levins eventually found the required language in loop analysis in a series of papers he published in the mid-1970s. Delving into loop analysis exceeds the scope of the present paper; the relevant point is not the solution (loop analysis) but the thing it meant to solve (niche construction). That Levins was concerned with the absence of an appropriate mathematical language for niche construction indicates that in the late 1960s and early 1970s, Levins was occupied with the relations between organism and its environment, and specifically with formulating those relations anew.

### The evolutionary context

As mentioned in the introduction, one peculiar feature of The Strategy is the fact that none of the models that appear there were new. Levins’ use of models in The Strategy meant to demonstrate his notions concerning modeling (that would be adequate for population biology), but the models were originally published in his works on the meaning of “fitness” in a changing environment. While niche was the central ecological aspect for establishing evolutionary ecology, Levins’ perception of fitness was the central evolutionary aspect. In order to understand the novelty in his view of fitness, I begin by briefly discussing the prevalent notion of fitness in the early 1960s, to which I will refer as the lock-and-key perception.

In its simplest version, the lock-and-key perception of fitness means that the environment poses challenges, and some organisms are equipped with better morphological traits to cope with them than others. Certain degree of variation with respect to given traits is always present in a population. Under conditions of environmental challenges (limited resources being central) some of these variations would be advantageous and increase the organism’s chances of survival and reproduction. Other variations would hinder those chances, and others still will have a negligible effect, or no effect at all. The influence a variable trait has on the organism’s chances of survival and reproduction does not derive from innate qualities of any variation, but from the way different variations influence the organism’s ability to operate within a particular environment. Different habitats favor different features. In a given environment, individuals with the beneficial variation (in that environment) will be more likely to survive to reproduction age, as well as gain better access to means of reproduction (for sexually reproducing species) and thus will produce on average more offspring. Thus, assuming that morphological traits are genetically inherited, the beneficial variation will become more prevalent in the population. Organisms equipped with beneficial variations are considered to have “higher” fitness than those who are not equally equipped. In this view, fitness is the factor that differentiates between organisms that do not survive to reproduction age, organisms with low reproduction rate and organisms with high reproduction rate. Thus, “fitness” does the discriminatory work required for natural selection to operate in a non-random way.

That portrayal of the lock-and-key view is highly schematic, but delving into the conceptual difficulties of this view exceeds the scope of the current paper. See (Abrams, 2009; Ariew & Lewontin, 2004; Matthen & Ariew, 2002; Mills & Beatty, 1979; Millstein, 2002; Walsh et al., 2002) for a more comprehensive account. The important point for the moment is that Levins’ perception of fitness stands against this background; while the lock-and-key view assumes environmental stability, environmental heterogeneity is a central assumption in Levins’ view. In his doctoral work, Levins enumerated several environmental and organismal properties as parameters that determine the optimal strategy for survival and reproduction; of those, two ordered pairs are particularly important: first, the grain (environment) and the fitness set (organism); second, uncertainty (environment) and information-receiving capacity (organism). The choice of these ordered pairs indicates that in Levins’ view organism and environment stand in co-evolutionary relations. Heterogeneous environments challenge organisms largely due to inherent uncertainty. Hence, understanding Levins’ view of the organism-environment relations should begin with uncertainty (Levins, 1968a, p.11):

There is always the danger that by the time the adaptation has taken place the environment has changed. This danger is of course greater when the delay is longer, and is especially so when the population adapts from generation to generation by genetic change through response to natural selection. [Q14]

Accordingly, the crucial aspect of the organism’s ability to handle the environment is not its morphological properties, but its ability to devise a coping strategy. Behavioral plasticity becomes central under conditions of changing environment; however, in certain instances, the organism’s coping strategy might also involve altering one or more morphological property (Levins, 1968a, p. 13):

The organism is conceived of as receiving this information [relating to environmental changes] through several receptor systems or through its whole body, processing this information into a prediction of future environments, and responding to this prediction with the formation of that phenotype which is the best strategy for the predicted range of environments. There is no necessary relation between the physical form of the information and the environmental factor to which the organism is responding. What is important is the statistical relation between them. [Q15]

This quotation can give the impression that Levins thought that organisms are able to predict future environments and make morphological adjustments according to the requirements that the predicted environment will pose. This impression is based on insights that derive from two lines of theoretical works in biology, namely, agency of organisms (Arnellos & Moreno, 2015; Barandiaran et al., 2009; Kauffman & Clayton, 2006; Nadolski & Moczek, 2023; Newman, 2023; Walsh, 2015), and phenotypic plasticity (Ehrenreich & Pfennig, 2016; Forsman, 2015; Gilbert et al., 2015; Moczek et al., 2011; Sultan, 2015; Uller et al., 2020).^11^ Writing before both these lines of work, Levins could not have dealt (or scientifically explain) either phenotypic plasticity or the agency of organisms. While not being able to deal with these issues in the same way they are addressed in recent years, it was still important to Levins to at least consider them as theoretically possible.

There are two crucial differences between the lock-and-key perception and the view advocated by Levins; first, a prominent implication of a variable environment is that a morphological trait has more than one point in the environment that it must “optimize” to. Second, the organism in Levins’ view, is not construed as passive. It is not morphology that allows the organism to survive, but its ability to predict environmental changes correctly and prepare strategically to the challenges that they pose. While such strategy may involve morphological changes, plasticity is more readily attributable to behavioral traits than to morphological ones. In a changing environment, optimizing a morphological trait to one set of environmental conditions could be lethal in the following set of conditions. Thus, and this is the important point, it is the strategy for coping with environmental changes (rather than the morphology) that is being selected. The agential language should not be taken literally; while Levins did not adhere to standard (at the time) optimality arguments,^12^ to the extent that there is an optimizing “agent” in his view, that agent is (probably) natural selection.^13^ Put differently, even in those situations where an organism alters its morphology, it should not be seen as deriving from an informed decision-making process.

Levins’ very definition of fitness reverses the direction of the relations between phenotype and environment. While the lock-and-key view conceives fitness as optimization of the suitability of a phenotype to a given environment, in Levins’ notion of fitness, the phenotype is seen as stable, while the environment changes; accordingly, the important factor for fitness becomes the ability to alter the environment to increase its suitability to the requirements posed by a given phenotype (Levins, 1968a, p. 13). Selection does not operate on morphological traits, but on the ability of organisms to adjust their environment. This can be done in several ways:

1. Move to a more hospitable environment (hence the centrality of the term “grain”).
2. Change the environment such that surviving with a given morphology becomes possible.
3. The theoretical possibility of changing the morphology.

The change of perspective between the two notions of fitness is noteworthy; while this is not niche construction in the accepted sense,^14^ it subverts both the passiveness ascribed to the organism and the assumption of environmental stability. Moreover, the organism may choose the environment, but if a changing environment is chosen, the organism must survive in all its forms. Not surviving a season means not surviving. That leads us to the fitness set, which is the relation between fitnesses in different environments for each potential phenotype (Levins, 1968b, p. 17). In a variable environment, different environmental expressions require different morphological or behavioral expressions, and it is not always possible to alter between them. The overall fitness in such situation depends on the fitnesses in the separate environments, (Levins, 1968b, p. 18):

The notion of grain comes of course from the size of patches of environment. If the patch is large enough so that the individual spends his whole life in a single patch, the grain is coarse, while if the patches are small enough so that the individual wanders among many patches the environment is fine-grained. [Q16]

The fitness set is but one element of an optimal strategy; the second element is the tolerance of each phenotype, i.e., the ability to function while not in the optimal environment for the phenotype (and Levins stresses that in certain circumstances this ability to function does not mean much more than sheer survival) (Levins, 1968b, p. 17). The final element of an optimal strategy is the level of uncertainty which is correlated with the grain: the more fine-grained the environment, the lower the level of uncertainty within that environmental grain. The correlation between size of grain and the level of uncertainty can be explained both from the side of the environment and from the side of the organism.

From the side of the environment, fine-grained patches are less likely to alter; in fact, the limits of a fine-grained patch could be defined as the limits within which the patch in question does not change, or changes in restricted ways. The levels of uncertainty in a patch defined in this way are quite low. From the perspective of an organism, as discussed in Sect. 4.3, granularity measures the rate of environmental changes. All other things being equal, an organism that encounters multitude of environments is better adapted to changes than an organism that stays a significant portion of its life in a given environment. While not mutually exclusive, Stability (of the environment) and certainty (of survival) should not be seen as synonymous. It could be argued that the ability to cope with instability provides certainty of survival.

To conclude this section, according to Levins, the meaning of fitness is an optimization process, not of a morphological phenotype but of a survival strategy. This strategy is meant to cope with uncertainty posed by a varying environment (and becoming more severe in a coarse-grained one); in order to do so, the organism may choose the environment that is best suited to its morphology, its ability to change the morphology, and the level of tolerance that its morphology allows.

### Modifying nature

On top of modeling desiderata, The Strategy enumerates several modeling goals, specifically, understanding, predicting, and modifying nature. That modifying nature is a valid objective of a modeler could be seen as another peculiarity of The Strategy; moreover, this view raises some conceptual difficulties: first, what does it mean that models can be used to modify nature? Second, what separates natural modifications with modeling from other methods of natural modifications? Finally, in what sense does that goal overlap with the others? While the third question exceeds the scope of the current paper, addressing the first two will connect the discussion concerning the relations between fundamental and applied ecology (Sect. 3) with Levins’ general approach to modeling.

Given Levins’ definition of “fitness” as the strategy of an organism to cope with heterogenous environment (which includes active modification of said environment), modifying nature is the very thing that organisms do and organisms at all levels engage in such modifications. Moreover, that definition of fitness relates not only to the organism’s ability to modify its surrounding, but also to its ability to predict the environmental modification that would be most beneficial to its survival. In this sense, the overlap between modification and prediction is not unique to modeling. Finally, the fact that modeling is a purposeful behavior does not render that practice different from any other form of natural modification. True, the modifications that derive from modeling are premeditated; however, all modifications of nature could be seen as goal-oriented behavior.

The previous paragraph does not attempt to argue that Levins adhered to the notion that model formulation is somehow akin to niche construction; there is no evidence that Levins accepted such a view. Instead, the previous paragraph means to highlight the following question: given that natural modification is a set of phenomena that takes palce on several levels, in what sense are models difference-makers with regard to these phenomena? How are natural modifications done with models different from modification done without models?

The use of models for the purpose of natural modification is the point where Levins’ rejection of the dichotomy between fundamental and applied science (discussed in Sect. 3), his view on environmental preservation and the need for ecological interventions (Sect. 4.1) and his skepticism towards the managerial ethos of ecosystem ecology (Sect. 4.2) come together. The distinction between model-informed modifications from other methods of modification relates to the normative aspect of the managerial ethos towards interventions. Within this framework, modifying nature through model-formulation is the stance that models can help us modify nature in informed ways in order to achieve specified goals. Under this definition, the use of models is predicated on divisions that are inherently political, among them the division of authority (who gets to decide the purposes); the division of labour (who does the modeling and who does the actual modification); the division of resources that derive from management of nature (who benefits); and the social and environmental implications that derive from these activities (who must become a climate migrate). However, two divisions relate to scientific practices and their epistemic values.

As seen in Sect. 4.1, environmental degradation was a central concern for ecologists from the late 1950s, with the implication that ecology as a discipline should be active in formulating public policies aiming at preservation. Even ecosystem ecology, the theoretical program for analyzing the dynamics of the flow of energy within a community, was partially motivated by such real-world concerns. Ecosystem ecology was formulated, in part, so that conservation policies could be informed. However, there are different standards of “informed” that could treat the same policy differently. During the 1960s, one disagreement over epistemic standards concerned the use of computer simulations and numerical solutions to formal models. In 1968, Kenneth F. Watt published his textbook *Ecology and Resource Management*. That work advocated ecosystem ecology as an applied approach to handling real-world ecological problems and specifically how best to use natural resources while minimizing environmental degradation. In the introduction to the book, Watt stated its two objectives: to present a general theory of resource management, and to advocate the use of simulations as a way to put ecosystem ecology into practice (Watt, 1968, p. 3). In a paper on complex systems from 1970, Levins stated his conception of the goal of science (Levins, 1970b, p. 78):

I would assert that the goal of prediction is a subordinate one: a contingent objective which is legitimate only to the extent that it helps us decide among alternative hypotheses, or in situations where there are applied problems of importance, but not equivalent to the goal of science itself, which is explanation. [Q17]

Levins’ approach to modeling complex systems (and its contrast with the approach he ascribed to Watt) will be elaborated on in Sect. 5.2. As for the meaning of “explanation”, Levins never gave an explicit account of what constitutes explanation (in his view), but certain points can be deduced. It should be noted here that Levins and the computation approach adhere to a different standard of being “informed”. Section 5.3 will address the relations between computation and the state of being informed more thoroughly, but for the moment, it should be noted that the computation approach focuses on the availability of certain instruments (numerical techniques, made available with computers) and the deployment of these instruments for solving problems. Levins’ approach, on the other hand, focuses on the epistemic accessibility of those instruments to their users. In Levins’ view, a decision cannot be considered “informed” if it is based on a model that we do not understand, even if it is possible to solve the model numerically.

## Discussion: Levins’ view of modeling, contextualized

### Instrumental view of models

A good starting point for the discussion on Levins’ general approach to modeling would be his notion of the meaning of “models”. What sort of entities, in Levins’ view, are considered to be “models”? The philosophical literature concerning scientific models is vast and covers a broad range of topics. This assertion is easily demonstrated by checking the bibliography section in (Frigg & Hartmann, 2020). However, most items in that bibliography were published after The Strategy and cannot elucidate the question at hand. Still, Levins’ own words from a paper on complex system could help answering the question (Levins, 1970a, p. 75):

Suppose that we did know the interrelations among all parts of a system and would describe the rate of change of each variable as a function of the others. Then we would have a very large set of simultaneous non-linear equations in a vast number of variables […] These equations will usually be insoluble. They would be likely to be too numerous to compute. If we could compute, the solution would simply be a number. If we could solve the equations the answers would be a complicated expression in the parameters that would have no meaning to us. Therefore the only way to understand a complex system is to study something else instead. […] That something else is a model. A model is a theoretical construction, a collection of objects and relations some, but not all, of which correspond to components of the real system. In one sense it is a simplification of nature. [Q18]

Three points could be drawn from this quote: first, that it does not offer a definition to the term “model” beyond *theoretical construction*. It seems that the pressing question for Levins is not what a model *is* but how a model is *used*. Levins considered models as epistemic instruments.^15^ Second, the use of model as presented here seems akin to what modern philosophical literature labels “surrogative reasoning” (Contessa, 2007; Plutynski, 2006; Swoyer, 1991). Finally, while it is possible to use models for any number of reasons, one central objective for deploying those theoretical constructions (according to Levins) is to cope with complex systems. Combined, the second and third points mean that models are instruments that could be used for reasoning about a different system that is not directly accessible otherwise.

Levins thought that a given scientific discipline has a set of common practices, standard tools and technical abilities (including its use of mathematics); however, one approach that Levins identifies as common across disciplines is reductionism (Levins, 1970a, p. 73). Levins believed that the scope of problems that reductionism can solve is limited (Levins, 1973a, pp. 112-113), and that sooner or later every research program is bound to encounter complex phenomena, where “complexity” means that such phenomena cannot be reduced to a given set of constituent elements (Levins, 1973b, pp. 113-114) or that the reduction distorts the target system to the extent that the surrogative reasoning about it is invalid. Levins considered modeling as the appropriate approach to describe and analyze the dynamics of complex systems, and they become prevalent when a research program encounters complex phenomena (Levins, 1973a, p. 114).

A comprehensive discussion about Levins’ works on complex systems exceeds the scope of the current paper; however, several themes tend to repeat themselves within that body of work. One of these themes is that the analysis of a complex system produces epistemological rather than ontological results (Levins, 1974, p. 126). There is no appropriate way to divide a complex system into subsystems; accordingly, when analyzing such a system, the results can only be correct within the decomposition that the researcher determined. When considering that models are epistemic instruments meant to deal with complexity, the meaning of the terms used in The Strategy to describe sufficient parameters (suitable, containing the relevant information, etc.) becomes clear: the sufficient parameters are sufficient for the model to perform the task that the researcher assigned to it within the framework of research.

Complexity seems like the inevitable result of integrative projects such as population biology and [Q2] demonstrates the specific challenge that Levins associated with that program. To be clear, simultaneously addressing the theoretical questions concerning the relations between an organism and its environment (as seen in Sect. 4.3) and the questions concerning the meaning of fitness in a changing environment, especially one that the organism takes part in changing (Sect. 4.4), brings about the kind of complexity that [Q2] describes. An attempt to directly handle this kind of a system is bound to encounter the same pragmatic difficulties described in the beginning of [Q18], namely, *a very large set of simultaneous non-linear equations*. Such an attempt amounts to the brute force approach that Levins referred to in The Strategy, and the next section will contrast that approach with the one Levins advocated.

The Strategy applies these general ideas of modeling to the context of population biology. As seen in the second section, The Strategy begins with enumerating methodological difficulties for the integration of population genetics, population ecology and biogeography into a single field of population biology. The previous section elaborated on the conceptual difficulties associated with this effort. As The Strategy makes clear, Levins’ approach to dealing with such difficulties relied heavily on the deployment of models, namely, formulating abstract representations of the scrutinized systems and treating the representation as a surrogative reasoning system.

### The intelligibility requirement

Understanding models as epistemic instruments for coping with complexity, some approaches to modeling are bound to be more productive than others. Indeed, Levins’ statement in The Strategy that “*The naïve*,* brute force approach would be to set up a mathematical model which is a faithful*,* one-to-one reflection of this* [population biology] *complexity*” (Levins, 1966, p. 421) highlights an approach to modeling that Levins rejected. In a review of Watt’s, 1968 book mentioned above, Levins enumerated some of the practical difficulties he associated with a complete model with one-to-one correspondence to its target system (Levins, 1968a, p. 304):

The most general kind of model, involving many species with internal genetic, physiological, and age heterogeneities, will have a large number of components. The pairwise interactions of these components may be order of magnitude more numerous. Higher order interactions would be too numerous to handle. [Q19]

While Levins ascribes Watt the ambition to formulate such a general model with a one-to-one correspondence with the target system, he did not think that Watt had achieved something along such lines, and states that the approach in the book is *narrowed to a refinement of traditional population ecology which is interdisciplinary only in including cases from fisheries and entomology* (Levins, 1968, p. 304). However, the more important line of criticism was not that Watt failed to achieve models with one-to-one correspondence but that the ambition for such models is misplaced to begin with. That point does not relate to Watt or his book, but to a brute force approach to modeling.

While The Strategy demonstrates three desiderata that model makers aim to maximize in order to achieve certain epistemic goals, [Q19] seems to be making the following point: the reason that trade-offs between these desiderata are required (the reason to have a modeling strategy) is that there are also requirements from the resulting model. The closing paragraph of The Strategy enumerates different requirements, and different kinds of work that models could be used for, among them understanding and controlling nature (Levins, 1966, p. 431). As [Q1] highlights, some of these uses are contradictory (describing the dynamics of a highly complex system on the one hand while being intelligible on the other hand). Levins warned against formulating a model whose equations are not soluble (or their solutions are not interpretable) since such a model cannot perform the task assigned to it by the model maker (Levins, 1966, pp. 421-422). To be clear, this is not a modeling desideratum in the same sense as generality, realism, or precision; rather, this is an epistemic requirement that the resulting model must meet. In what follows, I will address it as the intelligibility requirement.

While the intelligibility requirement tends not to receive the same attention as the others, it is hardly an “implicit” one. Levins highlights the importance of intelligibility throughout The Strategy, and it seems to be the only requirement that cannot be sacrificed. Intelligibility is the requirement that necessitates trade-offs in model-building, and the following points highlight its centrality in Levins’ view: first, as Levins stresses (Levins, 1966, p. 421), adding a variable to a given model (say, for the sake of realism) means adding another equation that deals with that variable. Second, as seen in [Q19], the number of interactions between different components increases much faster than the number of new components. Taken together, these points convey that modifying a model in a manner that disregards our cognitive and technical restrictions can result in a very large set of equations that cannot be solved analytically at the same time, and even if they are soluble, the solution could have no meaning to the model user. The same review presents a term that Levins associated with brute force modeling, namely, universal model (Levins, 1968a, p. 304):

Of course it may be argued that the genetic differences, physiological states and meteorological patterns are not always important. This is precisely the point: a model which claims universality must leave room for all variables which are ever important. [Q20]

The meaning of “universality”, as implied here, is twofold: on the one hand, it refers to a single model that encompasses the full scope of its target phenomenon. On the other hand, that same model should be, in principle, applicable to numerous target systems. Michael Weisberg seems to hit the nail on the head (regarding Levins’ concerns) when pointing out that universal models tend not to generalize beyond a particular phenomenon (Weisberg, 2006a, p. 362). Levins’ alternative to the brute-force approach entailed the use of clusters of models. Each model in the cluster is highly idealized, sacrificing one desideratum in order to maximize the other two (Levins, 1966, p. 422). As discussed in the second part, such modeling approach requires certain artificial assumptions, and the resulting models are, accordingly, partial representation of their target systems. As [Q4] demonstrates, Levins was aware of the problem, and suggested clusters of models as a solution.

Clusters of models avoid the problems of brute-force modeling since they are meant, in Levins’ own words, to *treat the same problem*. A cluster of models does not generalize any more than a universal model but given the restricted purpose of a cluster, that fact is not problematic. A cluster of models simply does not claim universality. Moreover, [Q4] stresses that robustness is a feature of a cluster and not of any single model. The term “robustness” in The Strategy refers to our confidence that the results produced by a cluster do not derive from an unrealistic assumption made by one (or more) model in it. This, I believe, is the meaning in the phrase *our truth is the intersection of independent lies*. One implication of that phrase is that models in a cluster are treated discretely; every model may include non-linear equations, but the models should not be solved simultaneously. The standard of robustness that Levins adhered to relies on a comparison between results of different models; that means that no model is allowed to interfere with the dynamics treated by another model in a cluster.

The central part of The Strategy is titled “Robust and Non-robust theorems”. Given Levins’ training as a mathematician during his undergraduate studies, when he writes about theorems it is reasonable to understand the term as mathematicians do,^16^ i.e., as a statement that can be deductively proven from axioms and previously proven theorems. While “theorem” seems like a wrong term to describe a natural system, Levins insists on using it. The insistence to use “theorem” seems to derive from technical restrictions to test the theorems empirically, either due to technical difficulties (observations would require a long time), or because observations are inherently impossible. In this view, models become instruments for testing theorems. Clusters of models avoid the problems that Levins associated with universal models in two ways: first, as discussed above, when several models (with different assumptions) are used to test the same theorem and point to similar conclusions, the theorem is considered robust. A single model simply cannot establish the robustness of a theorem. Second, each model in the cluster can be highly simplified, and they do not operate at the same time, so the intelligibility requirement is met.

### What counts as being informed?

In Sect. 4.5 we have seen that resource management was a central concern for ecologists in the mid-1960s, and that ecosystem ecologists implemented computational methods for that purpose. Now it is the time to explain Levins’ suspicion of these methods. When Levins wrote The Strategy, he was affiliated with the University of Puerto Rico and did not have access to computers. However, his suspicion towards numerical solutions ran deeper than mere lack of access. A year after the publication of The Strategy Levins moved to the University of Chicago, where he started collaborating with Richard Lewontin. Yet, whereas Lewontin was a pioneer in deploying computer simulations (Lewontin & Dunn, 1960), Levins did not incorporate computers in his work until the mid-1970s (Levins et al., 1975).^17^ A draft from Levins’ archive indicates the reasons for his reluctance to use computational methods (Levins, n.d.):

The increasing power of computers allow for relatively easy numerical solutions to even highly complex problems, but does not usually provide an understanding of the reasons for the observed dynamics. Further, computational methods require a degree of precise specification of equations and parameters which is not accessible in these [ecosystem and epidemiology] fields of study. [Q21]

While using computational methods such as numerical solutions can produce predictions, these predictions do not meet, in Levins’ view, the epistemic standards that constitute understanding. In this sense, computational methods facilitate something that resembles a brute force approach, but they do not meet the standards required in [Q17] and thus should not be seen as an explanation. Not understanding the *reasons for the observed dynamics* means that we also do not understand the reasons for the predictions that the numerical solutions produce, and accordingly cannot know in advance the circumstances where such predictions will fail. These are general concerns about computational methods, but they have real-world implications, and the document mentions fields such as ecosystem and epidemiology as its target fields. When applied to natural resource management, will we consider a policy that is based on predictions that we do not understand “informed”?

Contrary to numerical, quantitative, solutions, deploying clusters of models with the standard of robustness that Levins adhered to is a qualitative approach. As seen in [Q5], in Levins favorite type-3 modeling strategy, there is a hierarchy to clusters of models. The parameters in general models are higher-level entities, that derive from an abstraction which is done for pragmatic consideration, namely, the amount of real-world variables that constitute those higher-level entities. This number of variables is reduced to a smaller set of sufficient parameters which are suitable for a given level of analysis. However, while sufficient parameters are a pragmatic approach to modeling higher-level phenomena, this approach has its shortcomings, information loss being the most notable, and auxiliary models may be required to deal with these shortcomings.

The important point is that type-3 models avoid the problems of brute-force by producing a qualitative description of the dynamics of their target system. A qualitative description of the target system could, in turn, be used in further research, for example, by generating a prediction about future behavior of the target system. Under these conditions, a given model (or a set of models) is not the final word when describing a given target system. Instead, there is a feedback relation between the target system, the model, and the process of its formulation.^18^

### Qualitative modeling

Having clarified Levins’ general approach to modeling, it is easier to understand his preference for type-3 models. This preference seems to derive from two complementary factors: first, Levins preferred qualitative analysis. Second, type-3 models embody a qualitative modeling strategy. Levins’ preference of qualitative analysis was not purely aesthetic, as seen in [Q3].

While Levins believed that theories should produce testable predictions, he also believed that when engaging with the kind of work that requires mathematical modeling, there is a certain order to go about (temporally and hierarchically): one should begin by formulating a theory, the theory should then generate testable predictions, these predictions should be subjected to examination within acceptable margins of error, and only then the model is to be refined to gain a better quantitative fit with the results. Reversing this order in an attempt to increase precision would only result in what Levins derogatorily described as “curve fitting” (Levins, 1968a, p. 302). Put differently, in Levins’ view, one should first know the trends of the analyzed system, and only then attempt to get some quantitative predictions from his theory. Hence the importance of the feedback relations between the target system, the model, and the process of its formulation.

To be sure, Levins recognized the importance of precision, but the consideration at the heart of The Strategy is the trade-offs between the different desiderata, and specifically the costs and benefits of these trade-offs. A modeling strategy is a coping mechanism meant to circumvent technical restrictions; for example, in the time The Strategy was written computer simulations were mostly absent, so models had to be studied analytically. Whenever the technical state of a discipline does not pose a restriction (so that there is no need for trade-offs between desiderata), a modeling strategy is simply not required. This is not to say that qualitative modeling has no costs; in fact, Kingsland amply demonstrates these costs (Kingsland, 1995, pp. 190-191):

The consequences of stepping back to catch the patterns was that precision was lost. Predictions were stated in terms of choices between alternatives, with several sets of alternatives operating at once. The choices were often between ideal types, comparable to the frictionless pulleys, conservative fields, ideal gasses, and perfect crystals of physics, […]. Even if the ideal types were not found in nature, it was interesting to see how real species departed from the ideal. [Q22]

Regarding the trade-offs required by ecological and evolutionary questions he was interested in, Levins was concerned with the gains and losses that derive from prioritizing one desideratum over another. In this terminology, and according to Levins’ views, sacrificing precision is the least “expensive” modeling strategy: first, precision is the most quantitative desideratum. Second, a quantitative approach is more strongly restricted by the technical state of a discipline. In the absence of modern computers (or when refusing to use them), there was a strong argument to be made for the study of general trends of a system. Third, there is a limit to what can be expected from a model. As discussed above, Levins thought that the complexity of ecological communities restricts our ability to predict their long-term trends; under this view, precision could stand in contrast to trustworthiness.

When modeling complex systems, all models are inadequate in the sense that they leave out many (if not most) aspects of their target systems (Levins, 1966, p. 430). However, these inadequacies can help flesh out the relevant aspects of the target system; where a partial model provides predictions from which the empirical results diverge, this divergence points to the presence of factors that the model did not take into consideration. This is the meaning that quantitative results *are only important in testing hypotheses* (Levins, 1966, p. 422). This point pertains to Levins’ preference of qualitative models; to the extent that the inadequacy of models could be leveraged to gain better understanding of the target system, this functionality is more evident for models that do not aim to produce precise predictions.

Finally, even if quantitative analysis were prioritized, there would be several restrictions upon it: a variable might be essentially qualitative, or not defined well enough for quantitative analysis (Levins, 1966, p. 421). Alternatively, even when the phenomenon in question is in-principle quantitative, the discipline studying it may lack the instruments for gathering relevant information. In such situations, insisting on a quantitative approach only prevents progression, whereas the cost of prioritizing qualitative analysis does not seem particularly high.

## Conclusions

The purpose of the present paper was to read The Strategy of Model Building in Population Biology in its immediate programmatic context, in order to better understand Levins’ general approach to modeling. Of course, modeling is a central aspect of The Strategy, but as seen in the first section, Levins’ general view of modeling (when addressed on its own) is also the source of many of the peculiarities of that paper. My assumption throughout the paper was that understanding the specific questions Levins was addressing–be they theoretical, such as the meaning of fitness in a non-stable environment, applied, such as natural resources management, or methodological, such as the appropriate standard of robustness when dealing with complex problems–could elucidate Levins’ “philosophy of modeling”, even though they were not directly related to modeling. Put bluntly, I think that Levins’ approach to modeling, as it appears in The Strategy, derived not only from conceptual concerns about model-formulation, but to some degree also from particular questions that arose in evolutionary ecology.

Having seen some central aspects of the programmatic context, I would like to conclude by highlighting certain points concerning the role that Levins assigned to The Strategy within that program. First, The Strategy was never meant to be a full account of either the ecological or the evolutionary difficulties faced with when trying to establish population biology. Rather, the paper was a layout of a methodological approach that Levins believed would eventually solve at least some of the difficulties.

Related to the first point, the second is the intelligibility requirement. Be the requirements from the model as they may, it cannot satisfy them if it is not intelligible to its user. While models can be formulated for various reasons, one prominent reason for Levins (and as a practicing scientist he was hardly unique in this regard) was to analyze real-life target systems. Specifically, Levins wanted his models to help him gain better understanding of ecological systems, evolutionary processes, and the relations between fitness, selection, and environment. Regarded as an epistemic instrument assigned with this role, a non-intelligible model is, almost by definition, counterproductive. When considering that Levins understood “intelligibility” to mean the ability to use a model such that the user could understand the results that the model produces, the previous statement is not as rigid as it seem on first sight. Put differently, a model that produces precise predictions (within acceptable margins of error) is fine, under the condition that the user can understand those predictions. If a model user cannot understand the results that a model produces, then deploying it would require some justification. The types of models that Levins discusses in The Strategy derive, at least in part, from this epistemic requirement. A modeling strategy is only required when cognitive restrictions must be circumvented.

Finally, Levins recognized that models have a pragmatic dimension in the sense that they can be used to guide collective action. This dimension, which also has a normative aspect, exceeds the epistemic questions that derive from The Strategy. Since the philosophical literature about The Strategy tends to concentrate exclusively on the epistemic questions, the pragmatic dimension was, by and large, neglected. There is some degree of irony to that neglect; while the literature concerning Levins’ paper invokes his political and ideological commitments, as I have tried to show in Sect. 4.5, the pragmatic dimension of The Strategy is also highly political.

## Footnotes

1 Some of the quotations will reappear in later parts of the current paper. To avoid repetition, I have numbered the quotations, such that when a quotation reappears, I will only state its number.

2 In the early 1980s Levins became aware of the fact, requested– and received– the investigation materials. These include over 200 pages of reports, spanning the years 1950–1958, and could be found in a folder titled “FBI Documents” in the first box of Richard Levins’ papers.

3 Richard C. Lewontin (1929–2021) was an American evolutionary biologist. After earning his Ph.D. at Columbia University as a student of Theodosius Dobzhansky, he became a leader in developing the mathematical basis of population genetics and a pioneer in molecular evolution. The collaboration between Levins and Lewontin spans a broad range of issues from theoretical biology to social commentary.

4 I thank Prof. Shaul Katzir for turning my attention to this issue.

5 According to Orzack and Sober, in an instantiated model, the range of possible values for each parameter is assigned.

6 The syllabi for courses in human ecology he taught at the Harvard School of Public Health in the late 1990s and early 2000s make good example. Some syllabi can be found in a folder titled “Human Ecology” in the seventh box of Levins’ papers.

7 The Strategy does not deal directly with ecological communities. However, that paper did mean to provide a methodological outline to dealing with certain features of complexity. Those features are not restricted to any specific domain.

8 Robert H. MacArthur (1930–1972) was a Canadian ecologist. After earning his Ph.D. at Yale University as a student of G. E. Hutchinson, MacArthur became a prominent figure in theoretical ecology in general and population biology in particular. Levins and MacArthur collaborated extensively on the theoretical topics described in the current section.

9 Parts of that work were published from 1962 onwards. Unless specified differently, when addressing Levins’ dissertation in the present paper, I will refer to the 1968 version, since it is more user-friendly.

10 I thank Prof. Deborah M. Gordon for highlighting this issue for me.

11 The list of works given here, from both lines of inquiry, are not meant to be exhaustive.

12 Such arguments tended to assume environmental stability.

13 At the very least, I did not find evidence that Levins seriously considered a different optimizing agent.

14 It should be remembered that Levins’ work on fitness predates the work of Odling-Smee by over 20 years.

15 Developing a full account of instrumental approach to modeling far exceeds the scope of the current paper.

16 I thank Prof. Paul Hoyningen-Huene for turning my attention to this point.

17 It should be noted that this document is not part of a study but a proposal to establish a center dedicated to studying complex health problems. The proposed center was supposed to build a large-scale computer simulation. It should also be noted that Levins collaborated with Lewontin in proposing that center.

18 Some would describe such a relation as “dialectical”.
